# Membrane contact sites and cytoskeleton‐membrane interactions in autophagy

**DOI:** 10.1002/1873-3468.14414

**Published:** 2022-06-14

**Authors:** Patrick J. Duckney, Pengwei Wang, Patrick J. Hussey

**Affiliations:** ^1^ Department of Biosciences Durham University UK; ^2^ Key Laboratory of Horticultural Plant Biology, College of Horticulture and Forestry Sciences Huazhong Agricultural University Wuhan China

**Keywords:** actin, autophagy, chloroplast, contact site, cytoskeleton, endoplasmic reticulum, membrane, mitochondria, plasma membrane, vacuole

## Abstract

In Eukaryotes, organelle interactions occur at specialised contact sites between organelle membranes. Contact sites are regulated by specialised tethering proteins, which bring organelle membranes into close proximity, and facilitate functional crosstalk between compartments. While contact site proteins are well characterised in mammals and yeast, the regulators of plant contact site formation are only now beginning to emerge. Having unique subcellular structures, plants must also utilise unique mechanisms of organelle interaction to regulate plant‐specific functions. The recently characterised NETWORKED proteins are the first dedicated family of plant‐specific contact site proteins. Research into the NET proteins and their interacting partners continues to uncover plant‐specific mechanisms of organelle interaction and the importance of these organelle contacts to plant life. Moreover, it is becoming increasingly apparent that organelle interactions are fundamental to autophagy in plants. Here, we will present recent developments in our understanding of the mechanisms of plant organelle interactions, their functions, and emerging roles in autophagy.

## Graphical abstract

This review outlines the known mechanisms by which plants regulate interactions between organelles, their functions and importance to plant life. We discuss how ongoing research of the NETWORKED protein family and their interacting partners continues to uncover novel mechanisms of organelle interaction, as well as the emerging roles for plant membrane contact sites in the regulation of autophagy.

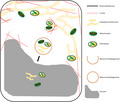

## Abbreviations

**AMCS**, actin‐membrane contact sites

**EPCS**, ER‐plasma membrane contact sites

**ERMES**, ER‐mitochondria encounter site

**E‐SYTs**, extended synaptotagmins

Eukaryotic cells are compartmentalised into specialised membrane‐bound organelles. Interaction between different organelles is fundamental to a range of processes including exchange of small molecules such as calcium, lipid transfer and autophagy [1, 2]. Physical Interaction between organelles occurs in various forms. For example, membrane ‘contact sites’ between organelles are mediated by protein complexes that tether membranes of each organelle in close proximity (approximately < 30 nm) without fusion of organelle membranes [1]. Conversely, organelle fusion is regulated by distinct tethering proteins, which facilitate the merging of organelle membranes [3]. Interactions between the actin cytoskeleton and organelle membranes are also mediated by specialised protein complexes with diverse roles [4], likely including regulation of organelle interaction.

In animal and yeast model systems, proteins that regulate organelle contact sites are well characterised, and interactions between virtually all organelles are known to be mediated by specific protein complexes. However, the identities of contact site proteins in plants have long remained elusive and, as such, the mechanisms of plant organelle interactions and their importance remained unknown. Orthologues of many yeast and mammalian contact site proteins are not present in plants. Having markedly different cell structures, it can be expected that plants have adapted unique toolkits of contact site proteins to regulate their unique functional niches.

Recent breakthrough discoveries have greatly advanced our understanding of how membrane interactions are regulated in plants. For example, the NETWORKED family of actin‐binding proteins have been characterised as dedicated plant‐specific contact site proteins, linking various organelle membranes to the actin cytoskeleton and mediating inter‐organelle interactions [5, 6, 7, 8]. The NETWORKED protein family has been a starting point used to uncover many novel contact site proteins. Continuing research has uncovered several plant‐specific mechanisms of organelle interaction, and their significance to plant life.

As we begin to understand the functions of plant membrane interactions, their importance in the regulation of autophagy is becoming apparent. During autophagy, cargo destined for degradation (including specific organelles and proteins) is encapsulated in vesicular membrane autophagosomes and targeted to the vacuole. It is now emerging in plants, as has been well characterised in mammals and yeast, that organelle interactions are involved in several stages of autophagy, including selective targeting of cargo for autophagy, autophagosome biogenesis, and transport and targeting to the vacuole.

In this review, we will discuss the discovery of the NET protein family and the subsequent characterisation of novel contact sites, as well as other known regulators of organelle interaction. We will present recent advances in the study of plant actin‐membrane contacts, organelle‐membrane interactions, and their roles in plant autophagy.

## The NETWORKED protein family

**Fig. 1:**
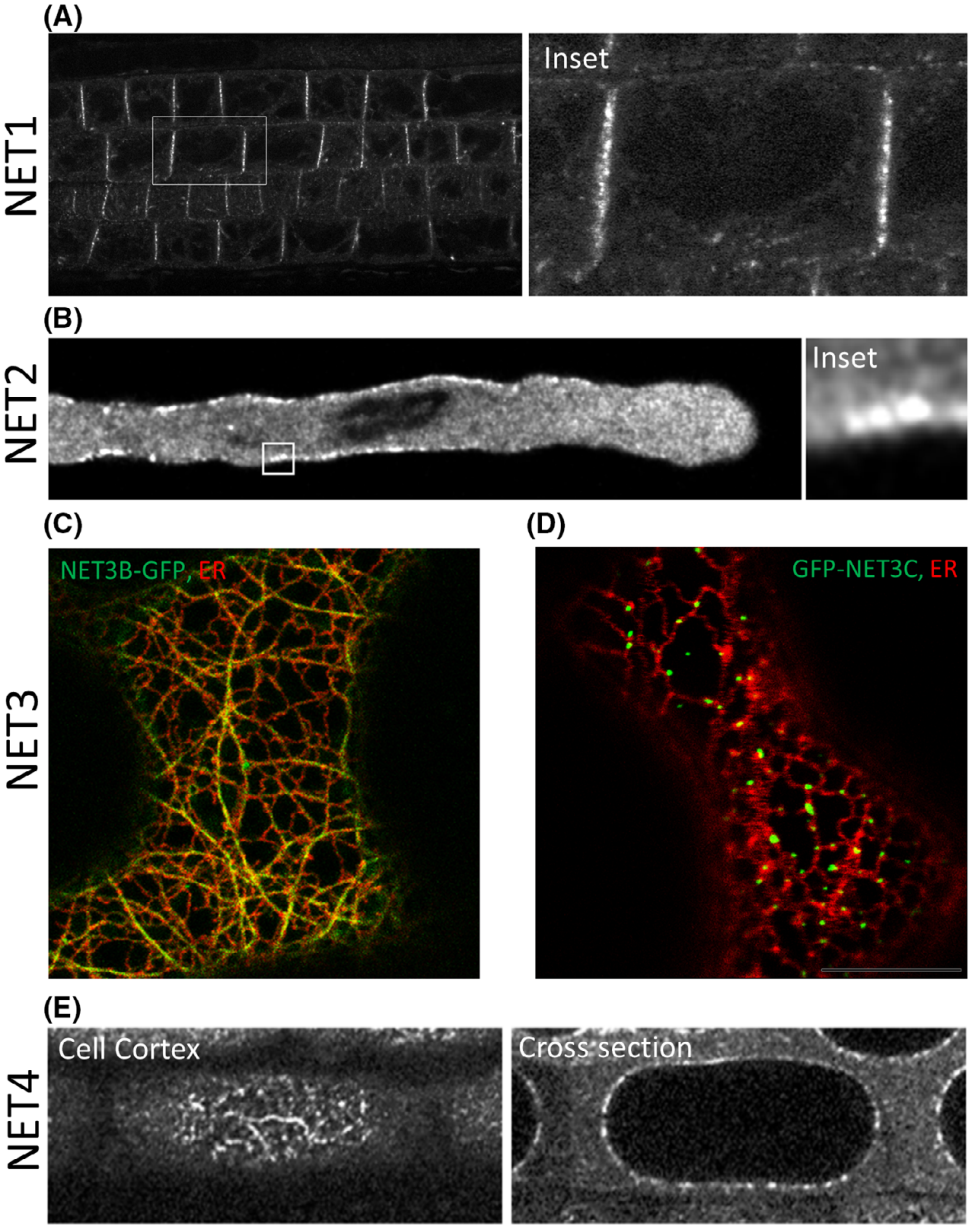
NETWORKED family proteins connect the actin cytoskeleton to specific membrane compartments. Depicted are the subcellular localisations of GFP fusions of representative NET proteins, analysed by confocal microscopy. (A) NET1A‐GFP predominantly localises to the transverse walls of root cell files, where it was found localised to plasmodesmata [5]. (B) NET2A‐GFP localises to punctae at the pollen tube plasma membrane [5]. (C) NET3B‐GFP binds actin to the ER [11]. (D). GFP‐NET3C localises to punctae at ER‐PM Contact Sites [5]. (E) NET4A‐GFP localises to punctae at the vacuole tonoplast [5]. Figure 1 has been reproduced from [5, 6, 11] (A), (B) and (E) have been reproduced from [5]; (C) has been reproduced from [11]; (D) has been reproduced from [6]. [Colour figure can be viewed at wileyonlinelibrary.com]

The NETWORKED (NET) superfamily of actin‐binding proteins has been identified as plant‐specific proteins, hypothesised to fill the functional niche of mammalian actin‐membrane linker complexes such as spectrin, filamin and α‐actinin, which are absent in plants [5]. The first NET family member was originally discovered in a protein localisation screen in a library of GFP fusions to Arabidopsis cDNAs [9]. One identified GFP fusion was named NETWORKED 1A because of the chimeric protein's localisation to a filamentous array that was confirmed to be the actin network. The cDNA fragment encoded a novel, NET actin‐binding (NAB) domain which was found to be conserved in 12 other Arabidopsis proteins and this domain then defined the NET superfamily of actin‐binding proteins. The NET protein family consists of four subfamilies, based on protein structural homology and expression patterns [5], and each bind F‐actin at a specific subcellular compartment. There exist four NET1 isoforms in Arabidopsis (NET1A‐D), of which NET1A binds actin and localises to the plasmodesmata (Fig. 1A). The pollen‐specific NET2 subfamily (NET2A‐D) binds the actin network at the plasma membrane (Fig. 1B) [5, 7, 10]. The NET3 subfamily meanwhile consists of three conserved proteins: NET3A has been observed to localise to the nuclear envelope [5], NET3B binds actin to the ER (Fig. 1C) [11] and NET3C binds actin at ER‐PM contact sites (Fig. 1D) [6]. Finally, the NET4 subfamily, consisting of NET4A and NET4B, both bind actin at the vacuole tonoplast (Fig. 1E) [5, 8].

**Fig. 2:**
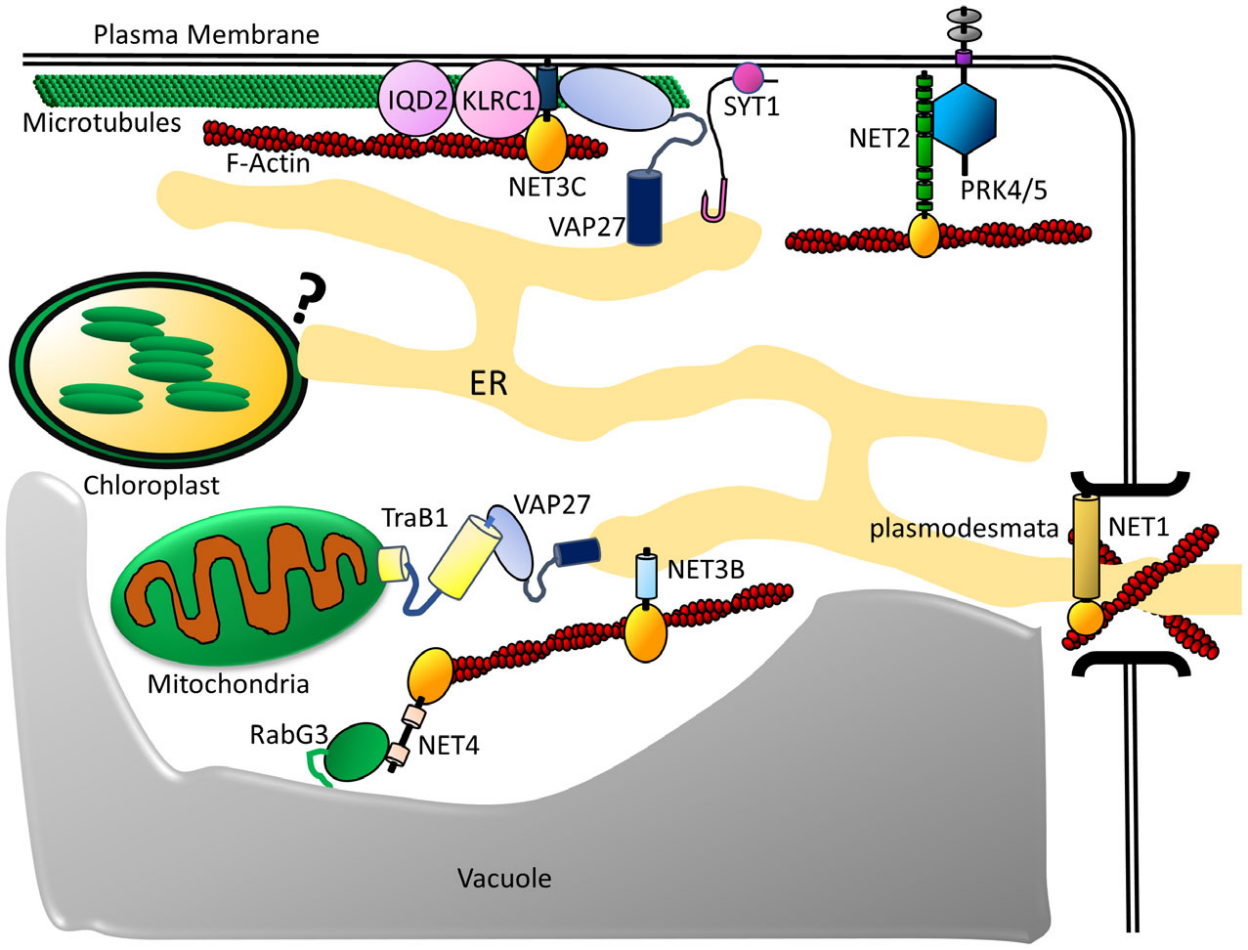
Characterised mechanisms of organelle interactions and actin‐membrane contact in plant cells. EPCS are mediated by SYT1 and VAP27. VAP27 binds to PM‐localised NET3C to connect the PM, ER and actin. NET3C forms a complex with the microtubule‐binding proteins IQD2 and KLRC1 to link the ER, PM, microtubules and actin. Actin‐PM contact sites are also mediated by NET2 and the transmembrane receptor kinases, PRK4&5. NET1 binds actin and localises to the plasmodesmata [5]. NET3B binds actin to the ER. ER‐mitochondrial interactions are mediated by the interaction of VAP27 with the outer mitochondrial membrane proteins, TraB1a and TraB1b. Interactions between the ER and chloroplasts occur through a yet‐uncharacterised mechanism. NET4 binds actin at the vacuole tonoplast through interaction with tonoplast‐localised RabG3. [Colour figure can be viewed at wileyonlinelibrary.com]

Therefore, the NET protein family represents a plant‐specific toolkit of proteins dedicated to linking the actin cytoskeleton to various membrane compartments. The characterisation of the NET family has established early examples of protein complexes that physically connect distinct organelles, through interaction of proteins localised to separate compartments. This has provided a starting point from which to expand our understanding of plant contact site regulation and function, as subsequent research into the NETs and their wider interactors continues to elucidate novel protein complexes involved in the regulation of organelle interactions. A summary of the NETs' interaction partners at membrane contact sites, described below, is shown in Fig. 2.

## Components of plant membrane contact sites

### Interactions between the cytoskeleton and plasma membrane

In mammalian models, proteins that tether the actin cytoskeleton to the plasma membrane, such as Spectrin, Filamin and α‐Actinin, are not present in plants [5]. Instead, plants possess several unique mechanisms of interaction between the plasma membrane and the actin cytoskeleton.

Recently, the NET2 subfamily of NET proteins was found to bind and anchor actin cables at the plasma membrane in pollen tubes through interaction with the integral membrane kinases, PRK4 and PRK5, at stable patches designated actin‐membrane contact sites (AMCS). These AMCS are important in the organisation of the cortical cytoskeleton and polar pollen tube growth [7, 10]. A direct link between a transmembrane signalling receptor‐kinase and F‐actin implicates a role for AMCS in extracellular signal transduction to the actin cytoskeleton to mediate actin‐driven subcellular responses to the environment.

Interactions between the actin cytoskeleton and PM are also mediated by NET3C, which binds F‐actin and is hypothesised to interact with the PM phospholipids through a putative C‐terminal lipid‐binding domain [6]. Further research on NET3C has revealed it to exist in a complex with the microtubule‐binding proteins, KLRC1 and IQD2, which together organise the cortical actin and microtubule cytoskeletons to regulate cell wall deposition [12].

### Endoplasmic reticulum‐plasma membrane contact sites (EPCS)

Contact sites between the ER and plasma membrane (ER‐PM Contact Sites; EPCS) have been extensively characterised in animals and yeast, from which canonical roles in inter‐organelle lipid transfer and calcium signalling have been attributed [13]. Perhaps, the best characterised EPCS proteins are the Vesicle‐Associated Membrane Protein (VAMP)‐Associated Protein (VAP) family; ER integral membrane proteins conserved across eukaryotes. The yeast VAP proteins, Scs2p and Scs22p localise to the ER to mediate tethering to the PM [14, 15] and interact with several PM‐localised ORP [Oxysterol Binding Protein (OSBP)‐Related Proteins] family lipid transfer proteins, including Osh2p and Osh3p, which facilitate lipid transfer between the PM and cortical ER [14, 16]. As in yeast, mammalian VAP also interacts with OSBP, and has also been shown to form EPCS through interaction with the PM‐localised Voltage‐gated potassium channel, Kv2 [17]. Mammalian Extended Synaptotagmins (E‐SYTs) also serve as tethering factors between the ER and PM; anchored to the ER membrane through an N‐terminal hydrophobic hairpin domain, and interacting with plasma membrane phospholipids with C‐terminal C2 domains [18]. Many such EPCS proteins, including VAPs, ORPs and E‐SYT proteins are conserved in plants, and their functions at membrane contact sites are beginning to emerge.

Following the discovery of the plant NET protein family, subsequent investigation of NET3C revealed it to mediate EPCS through interaction with VAP27‐1, which is a plant orthologue of mammalian VAP, and yeast Scs2 [6]. NET3C interacts with the ER‐integral VAP27 to tether the ER membrane to the PM, and overexpression of NET3C and VAP27 results in expansion of the EPCS [6, 19]. Plant VAP27 proteins have been shown to interact with endosomal clathrin and PI(4)P to maintain homeostasis of endocytosis [20], and NET3 and VAP27 have been shown to be important in normal plant development [6, 19, 21].

SYT1, the orthologue of mammalian E‐SYT proteins, has also been characterised in Arabidopsis, and has been shown to stabilise NET3C/VAP27‐mediated EPCS [22, 23]. SYT1 is important in the regulation of osmotic stress responses [24] and functions to enhance EPCS expansion under salt stress. This appears to be regulated by stress‐induced PI(4,5)P2 accumulation at the plasma membrane, with which SYT1 interacts through its conserved C2 domains [22, 25]

Our understanding of plant EPCS is still emerging, and the hypothesised roles of plant EPCS in processes such as calcium signalling or lipid exchange have yet to be characterised. Plant ORP proteins are likely candidates to mediate lipid transfer between the ER and plasma membrane; the Arabidopsis genome encodes 12 ORP proteins [26]; however, their functions have yet to be characterised.

### ER‐mitochondrial and ER‐plastid interactions

Interactions between ER and mitochondria are known to exist in plants [27, 28]; however, the protein complexes that mediate contact sites are poorly characterised compared to animals and yeast. In yeast, the ERMES (ER‐Mitochondria Encounter Site), mediated by a complex consisting of the ER‐integral Mmm1, and mitochondrial outer envelope proteins, Mdm10, Mdm34 and Mdm12, tethers the mitochondria to the ER and mediates lipid transfer between the two organelles [29, 30]. Additionally, the yeast mitochondrial import protein, Tom70, interacts with the ER‐localised lipid transfer protein, Ltc1, which also is likely to function in lipid transfer between the ER and mitochondria [31]. TOM70 also serves as an ER‐mitochondrial contact site protein in animals, and mediates ER to mitochondrial calcium transfer through recruitment of IP3R3 calcium channels [32]. In mammals, ORP5 and ORP8 are localised to ER‐mitochondrial contact sites, where they interact with the mitochondrial outer envelope protein, PTPIP51 and ER‐integral VAP proteins and are hypothesised to mediate lipid transport [33].

A novel protein complex mediating ER‐mitochondrial contact sites has recently been characterised in Arabidopsis. An interaction between VAP27 and the outer mitochondrial envelope proteins, TraB1a and TraB1b promotes interaction between the ER and mitochondrial membranes, and is important in maintaining normal mitochondrial morphology and respiratory function [21]. Additionally, plant ER‐mitochondrial interactions appear to have a functional role in mitochondrial dynamics: the mitochondrial outer envelope GTPase, Miro2, regulates tethering of mitochondria to the ER and promotes mitochondrial fusion [27]. It is also likely that plant contact site proteins regulate processes such as calcium and lipid transfer between the ER and mitochondria, as in animals and yeast. In particular, plant homologues of mammalian ORP5/ORP8 are promising candidates to mediate ER‐mitochondrial lipid exchange. However, such areas of research are yet to be fully explored.

The ER is known to reside in physical contact with the chloroplast outer envelope [28, 34, 35], and this connection appears to undergo dynamic remodelling under stress [28]. ER‐chloroplast connections are also likely to occur at chloroplast stromules; tubular protrusions of the chloroplast envelope which run contiguous with ER, suggestive of some interaction [36]. Membrane contacts are important for lipid exchange between the ER and chloroplasts, which is facilitated by CLIP1 lipase, localised to ER‐chloroplast contact sites [37] and chloroplast‐localised TRIGALACTOSYLDIACYLGLYCEROL (TGD) proteins [38]. Although the importance of ER‐chloroplast interactions is well understood, it is still unclear how the two organelles are physically linked, and remains an exciting area of future research.

### Actin‐membrane interactions at the vacuole

Contact sites between the actin cytoskeleton and vacuole have also recently been characterised in plants, and are important in regulating vacuole morphology [5, 39]. This may indicate an involvement of actin in regulating tonoplast remodelling, or vacuole fusion. The NET4 subfamily of actin‐membrane contact site proteins is emerging as a regulator of actin‐vacuole contact sites. NET4 proteins bind actin at the tonoplast [5, 8, 40], and may function to regulate vacuolar occupancy [40]. NET4 proteins have recently been characterised as downstream effectors of RabG3, which recruit NET4 to the tonoplast to mediate actin‐driven vacuolar remodelling in response to extracellular pathogen perception [8]. It is possible that actin may provide the driving force necessary to remodel the structure of the tonoplast, and the exact mechanism by which NET4‐mediated contact sites can regulate tonoplast morphology remains a focus of future research.

## The role of membrane interactions in autophagy

It is becoming increasingly apparent that plant membrane interactions have a crucial role in the regulation of autophagy. Inter‐organelle membrane contact is fundamental to many steps of autophagy progression. During the biogenesis of the autophagosome, autophagosomal membranes are generated from organelle membrane sources such as the ER, facilitated by an actin‐driven force [41]. Selective autophagic degradation of specific organelles requires their docking to the expanding phagophore which is a double membrane emanating from the ER and engulfs autophagic cargo to form the autophagosome [42]. The mature autophagosome is targeted to the vacuole through association with actin transport networks, and must fuse to the tonoplast to deliver its contents for degradation in the vacuole [42]. Therefore, protein regulators of organelle interaction can be expected to have important roles in the regulation of autophagy. Here, we will explore the potential roles of characterised contact site proteins in autophagy.

### Membrane contact sites in autophagosome biogenesis

The ER is one of the main sources of autophagic membranes, and ER‐membrane contact sites are known to have important roles in autophagosome biogenesis. In mammals, the EPCS proteins, VAPA and VAPB have been shown to regulate expansion of phagophores through the interaction of autophagy regulatory proteins, ULK1 and FIP200 [43]. Furthermore, ESYT‐mediated EPCS are necessary for localised PI3P synthesis at the expanding phagophore, which is a crucial first step in autophagy initiation [44]. Mammalian ER‐mitochondrial contact sites too are important in autophagosome biogenesis. Recruitment of ATG14, a regulator of PI3P synthesis, suggests that ER‐mitochondrial contact sites may have a role in localised PI3P accumulation at the phagophore [45].

In plants, VAP27‐mediated EPCS also appear to have an important function in autophagosome biogenesis, and *vap27* loss of function mutants exhibit autophagy‐defective phenotypes [46]. Plant EPCS are a site of autophagosome biogenesis, and bring together the ER membrane, cytoskeleton and plasma membrane‐localised endocytic machinery to drive formation of the autophagosome. This process is mediated by a complex of VAP27, the actin‐binding protein EH/Pan1, the T‐PLATE complex and clathrin [46]. It is possible that one role for VAP27‐mediated EPCS is PI3P synthesis at the expanding phagophore, as pharmacological inhibition of PI3P synthesis disrupted EH/Pan1 localisation to the autophagosome [46].

**Fig. 3:**
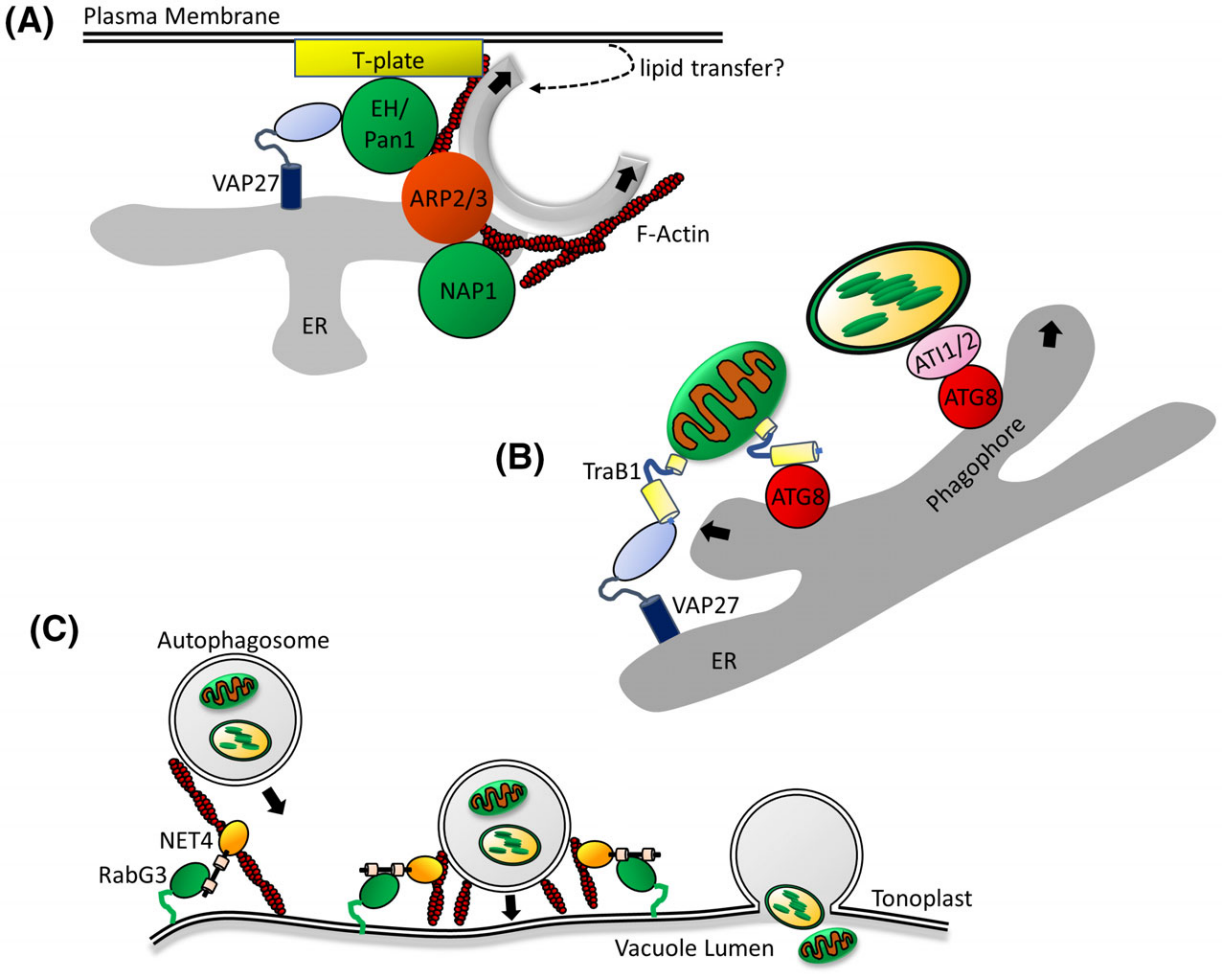
Potential roles of organelle interactions and actin‐membrane interplay in plant autophagy. (A) EPCS contribute to autophagosome biogenesis. The ARP2/3‐regulator, EH/Pan1 interacts with VAP27 and PM‐localised T‐PLATE components. Regulation of the cortical actin cytoskeleton and endocytic machinery functions to drive expansion of the ER‐derived phagophore. It is also possible that EPCS are important in lipid transfer from the PM to the phagophore membrane. Additionally, the SCAR‐WAVE protein, NAP1, promotes ARP2/3‐dependent actin dynamics at the ER to drive autophagosome biogenesis. (B) Organelle interactions with the ER may play a role in selective autophagy. The outer mitochondrial membrane proteins TraB1a and TraB1b bind VAP27 to mediate ER‐mitochondrial interactions, and mitophagy through interaction with ATG8. Tethering of mitochondria to the ER may promote their sequestration to the ER‐derived phagophore. ATI1 and ATI2 may also have a role in mediating interaction of the chloroplast and the ER or phagophore, and interact with ATG8 to mediate autophagic chloroplast degradation. (C) Actin‐Membrane interactions may function to target autophagosomes to the vacuole. NET4 localises to the tonoplast through interaction with RabG3, and may link actin transport networks to the tonoplast to promote autophagosome delivery. Regulation of actin‐driven force by NET4 may also promote fusion of autophagosomes and the tonoplast. [Colour figure can be viewed at wileyonlinelibrary.com]

Interaction of the actin cytoskeleton and ER is also important in autophagosome biogenesis in plants. The EH/Pan1 yeast orthologue activates the ARP2/3 complex to regulate actin polymerisation and drive membrane invagination during endocytosis [47, 48]. Arabidopsis EH/Pan1 interacts with the ARP2/3 complex at the autophagosome, and is likely to regulate actin polymerisation to drive autophagosome biogenesis [46]. ARP2/3 is also regulated by the SCAR/WAVE complex, members of which also regulate autophagosome biogenesis. For example, the SCAR/WAVE protein, NAP1 localises to nascent autophagosomes associated with the ER, and is important in autophagosome biogenesis [49]. These recent findings indicate that regulation of actin dynamics at the ER is an important step in autophagosome biogenesis. It is hypothesised that F‐actin may provide the driving force to shape the expanding phagophore, or connect the newly formed autophagosome to actin transport networks [49] (Fig. 3A). Interestingly, it has been demonstrated that disruption of the actin cytoskeleton does not inhibit bulk autophagy in plants, suggesting that autophagosome biogenesis and targeting to the vacuole can also be mediated by other mechanisms independent of actin‐membrane interactions [50] Recent findings indicate that plant ER‐mitochondrial interactions also may have a role in autophagy. The ER‐mitochondrial contact site proteins, TraB1a and TraB1b, have been demonstrated to interact with the autophagy protein, ATG8 through ATG8‐Interacting Motif (AIM) domains, and regulate autophagic degradation of mitochondria [21] (Fig. 3B). Further investigation of this link between plant ER‐mitochondrial interactions and autophagy may elucidate potential mechanisms by which these contacts may regulate autophagosome biogenesis.

### Plant ER‐organelle interactions and selective autophagy

Selective autophagy is the targeted autophagic degradation of specific cell components, including organelles such as mitochondria, chloroplasts, peroxisomes and the ER [51]. Organelles and other components targeted for autophagic degradation must be sequestered to the ER‐derived phagophore [52]. Therefore, interactions between organelles and the phagophore, and perhaps the ER, is inherent to their degradation by the autophagy pathway.

In yeast, it is known that ER‐mitochondrial contact sites, mediated by the ERMES complex, are required for mitophagy, likely to facilitate sequestration of mitochondria by the ER‐derived phagophore [53]. In plants, a similar role may be played by the ER‐mitochondrial contact site proteins, TraB1a and TraB1b. TraB1 proteins are important in autophagic degradation of damaged mitochondria under stress, and promote mitophagy by interacting with ATG8, perhaps targeting mitochondria to the nascent autophagosome [21]. TraB1‐mediated tethering of mitochondria to the ER may be important to supply ER‐derived membrane to the expanding mitophagosome.

Chloroplasts are also known to be degraded by autophagy [54], and it is possible that tethering between the ER and chloroplasts may also be an important step in the initiation of chlorophagy. ATG8‐interacting protein 1 and 2 (ATI1 & ATI2) are known to be important in stress tolerance, exhibit a dual localisation to the ER and chloroplast, and have a putative role in autophagic degradation of chloroplasts [55]. It is possible that ATI1 and ATI2 may serve to establish a tether between the ER and chloroplasts during chlorophagy.

Selective autophagic degradation of the ER, or ER‐phagy, also occurs in plants [56], and is likely to depend on EPCS and actin. In yeast, the VAP27 homologues Scs2/Scs22, Pan1 and the ARP2/3 complex regulate ER‐phagy by connecting the cortical ER to endocytic pits to facilitate actin‐driven sequestration of ER domains to the phagophore [57]. In Arabidopsis, EH/Pan1 and VAP27 may play a significant role in ER‐phagy through regulation of EPCS, actin and the endocytic machinery.

### Autophagosome fusion with the tonoplast

Autophagy requires the fusion of autophagosomes to the tonoplast. The mammalian orthologue of RabG3, Rab7, is important in fusion of autophagosomes with the lysosome [58]. In Yeast, the RabG3 orthologue, Ypt7, localises to autophagosomes and recruits the HOPS (Homotypic Fusion and Vacuole Sorting) complex, which facilitates fusion with the tonoplast through activation of SNARE proteins [59]. In plants, a role for RabG3 in vacuolar targeting of autophagosomes is emerging. RabG3 isoforms localise to autophagosomes and interact with the autophagy regulatory protein, ATG8 [60, 61, 62], while pharmacological disruption of RabG3 function has been determined to impair targeting of autophagosomes to the vacuole, perhaps through blocking assembly of the HOPS complex [62]. As downstream effectors of RabG3 [8], NET4 proteins may also function in fusion of autophagosomes to the tonoplast. The actin cytoskeleton serves as an intracellular transport network and NET4 may serve to connect and tether actin to the tonoplast downstream of RabG3. This may facilitate targeting of autophagosomes to the vacuole, or perhaps promote fusion through regulation of actin‐driven force (Fig. 3C).

## Conclusion

Our understanding of plant membrane contact sites and their constituent protein complexes is rapidly advancing, and characterisation of NET contact site proteins has provided a starting point for the wider characterisation of novel mechanisms of organelle interaction. As plant membrane contact sites are becoming better characterised, their diverse functions are increasingly appreciated. In plants, inter‐organelle membrane interactions are implicated in extracellular signal transduction across membranes [7, 8, 10], membrane remodelling [8, 49], maintenance of organelle structure and organisation [10, 12, 21], stress response [25, 46, 49, 55], lipid transfer between organelles [37, 38], membrane fusion [27], cell wall deposition [15] and cell morphology [10, 15].

Several novel types of plant contact sites have roles in the regulation of autophagy. Interactions between organelle membranes are known to play significant roles in autophagosome biogenesis, likely by facilitating interplay between regulatory proteins located to specific organelles, and lipid transfer between organelle membranes [45, 46, 49]. Inter‐organelle interactions are fundamental to the sequestration of specific organelles to the phagophore during selective autophagy, and plant ER‐mitochondrial contacts have been shown to be important for mitophagy [21]. As the components and functions of plant membrane contact sites become elucidated, we will further understand how interorganelle interactions regulate specific autophagic processes.
